# Oncogenic mutations in adenomatous polyposis coli (*Apc*) activate mechanistic target of rapamycin complex 1 (mTORC1) in mice and zebrafish

**DOI:** 10.1242/dmm.012625

**Published:** 2013-10-02

**Authors:** Alexander J. Valvezan, Jian Huang, Christopher J. Lengner, Michael Pack, Peter S. Klein

**Affiliations:** 1 Cell and Molecular Biology Graduate Group, Perelman School of Medicine at the University of Pennsylvania, Philadelphia, PA, USA.; 2 Department of Medicine, Perelman School of Medicine at the University of Pennsylvania, Philadelphia, PA, USA.; 3 Institute for Regenerative Medicine at the Perelman School of Medicine at the University of Pennsylvania, Philadelphia, PA, USA.; 4 Department of Animal Biology, University of Pennsylvania School of Veterinary Medicine, Philadelphia, PA, USA.

**Keywords:** APC, Wnt, mTOR, mTORC1, Zebrafish, Colon cancer, Polyposis, GSK-3

## Abstract

Truncating mutations in adenomatous polyposis coli (*APC*) are strongly linked to colorectal cancers. APC is a negative regulator of the Wnt pathway and constitutive Wnt activation mediated by enhanced Wnt–β-catenin target gene activation is believed to be the predominant mechanism responsible for *APC* mutant phenotypes. However, recent evidence suggests that additional downstream effectors contribute to *APC* mutant phenotypes. We previously identified a mechanism in cultured human cells by which APC, acting through glycogen synthase kinase-3 (GSK-3), suppresses mTORC1, a nutrient sensor that regulates cell growth and proliferation. We hypothesized that truncating *Apc* mutations should activate mTORC1 *in vivo* and that mTORC1 plays an important role in *Apc* mutant phenotypes. We find that mTORC1 is strongly activated in *apc* mutant zebrafish and in intestinal polyps in *Apc* mutant mice. Furthermore, mTORC1 activation is essential downstream of APC as mTORC1 inhibition partially rescues *Apc* mutant phenotypes including early lethality, reduced circulation and liver hyperplasia. Importantly, combining mTORC1 and Wnt inhibition rescues defects in morphogenesis of the anterior-posterior axis that are not rescued by inhibition of either pathway alone. These data establish mTORC1 as a crucial, β-catenin independent effector of oncogenic *Apc* mutations and highlight the importance of mTORC1 regulation by APC during embryonic development. Our findings also suggest a new model of colorectal cancer pathogenesis in which mTORC1 is activated in parallel with Wnt/β-catenin signaling.

## INTRODUCTION

Colorectal cancer (CRC) is responsible for over 600,000 deaths annually worldwide (USCSW Group, 2013). Some 80% of sporadic CRCs result from mutations in adenomatous polyposis coli (*APC*) (Kinzler and Vogelstein, 1996; Cancer Genome Atlas, 2012). Patients with germline *APC* mutations develop familial adenomatous polyposis (FAP), which is marked by hundreds to thousands of adenomatous colon polyps and progression to invasive carcinomas (Groden et al., 1991; Kinzler et al., 1991; Miyoshi et al., 1992). *APC* mutations occur within a mutation cluster region (MCR) and result in expression of a truncated protein that lacks the C-terminal half (Miyoshi et al., 1992). Intestinal cells in humans, mice and rats with these mutations undergo loss of heterozygosity, thus initiating tumor development (Amos-Landgraf et al., 2007; Haramis et al., 2006; Kinzler and Vogelstein, 1996; Su et al., 1992).

As a negative regulator of the Wnt signaling pathway, APC is a core component of the degradation complex that mediates the turnover of β-catenin. *APC* mutations therefore stabilize and constitutively activate Wnt/β-catenin signaling, a key step in the development of CRCs (Korinek et al., 1997; MacDonald et al., 2009; Morin et al., 1997; Munemitsu et al., 1995). Overexpression of β-catenin in the colon leads to adenoma formation (Romagnolo et al., 1999) whereas knocking down β-catenin reduces adenoma size and frequency (Foley et al., 2008; Scholer-Dahirel et al., 2011). In addition, CRCs with wild-type *APC* frequently have stabilizing mutations in β-catenin (Morin et al., 1997; Polakis, 2000), providing compelling evidence for the role of β-catenin in colorectal carcinogenesis.

However, several groups have reported that nuclear localization of β-catenin, which is required to activate Wnt target genes, is infrequently observed in early adenomas of patients with FAP, sporadic human polyps and microadenomas in a rat model of FAP, despite *Apc* loss of heterozygosity (LOH) and elevated cytosolic β-catenin (Amos-Landgraf et al., 2007; Anderson et al., 2002; Bläker et al., 2004; Kobayashi et al., 2000). In addition, defects in intestinal differentiation were observed in zebrafish *apc^mcr^* mutants without detectable nuclear β-catenin or activation of a Wnt transcription reporter (Phelps et al., 2009b). Although the absence of detectable nuclear β-catenin in adenomas could also reflect the sensitivity of the detection methods, the observations have nevertheless led to the identification of additional steps required for nuclear translocation of β-catenin, including activation of Ras and Rac1 (Phelps et al., 2009b; Zhu et al., 2012).

These observations have also raised the possibility that additional effectors downstream of APC might contribute to *Apc* loss-of-function phenotypes (Phelps et al., 2009a; Phelps et al., 2009b). APC has Wnt/β-catenin-independent roles, including regulation of apoptosis, microtubule dynamics, regulation of retinoic acid biosynthesis and cell-cell adhesion (Hanson and Miller, 2005; Phelps et al., 2009a). We previously found that APC directly enhances the activity of glycogen synthase kinase-3 (GSK-3) (Valvezan et al., 2012). GSK-3 in turn negatively regulates mechanistic target of rapamycin complex 1 (mTORC1) (Inoki et al., 2006) and thus we found that APC, acting through GSK-3, suppresses mTORC1 activity in cultured cells (Valvezan et al., 2012). Because mTORC1 promotes cell growth and proliferation and is aberrantly active in many cancers (Laplante and Sabatini, 2012), we hypothesized that oncogenic *Apc* mutations might activate mTORC1 independently of β-catenin, and that this activation is important for *Apc* mutant phenotypes.

**TRANSLATIONAL IMPACT**

**Clinical issue**

Colorectal cancer is responsible for over 600,000 deaths annually. The majority of sporadic colorectal cancers are caused by truncating mutations in the tumor suppressor gene *APC*. Inherited *APC* mutations cause familial adenomatous polyposis (FAP); a condition in which patients develop hundreds of intestinal polyps, some of which inevitably progress to cancer. The oncogenic effect of *APC* mutations has been largely attributed to the role of APC protein as a negative regulator in the Wnt/β-catenin signaling pathway; however, the contribution of other downstream effectors cannot be ruled out. Identification of additional downstream factors that meditate the effects of *APC* mutations could lead to effective therapeutic approaches to treat colorectal cancers and FAP. Despite its importance, the biochemical functions of APC are not well understood, so the molecular consequences of oncogenic *APC* mutations remain enigmatic.

**Results**

Here, the effects of oncogenic *Apc* mutations were examined in zebrafish and mice. mTORC1, a nutrient sensor that promotes cell growth, is shown to be strongly activated in *apc* mutant zebrafish and in intestinal polyps in *Apc* mutant mice. Furthermore, mTORC1 inhibition attenuates developmental defects caused by *apc* mutations including early lethality, circulatory defects and liver enlargement. Crucially, combined inhibition of mTORC1 and Wnt/β-catenin signaling rescues aberrant body curvature in *apc* mutant zebrafish, which is not rescued by inhibition of either mTORC1 or Wnt signaling alone.

**Implications and future directions**

This study establishes mTORC1 as a crucial, β-catenin-independent effector downstream of oncogenic *Apc* mutations. These data demonstrate that APC suppresses the activity of mTORC1, and that oncogenic *Apc* mutations activate mTORC1. The authors demonstrate that mTORC1 activation is essential for mediating the effects of *Apc* mutation in zebrafish development, given that mTORC1 inhibition ameliorates the associated developmental defects. These findings support a new model in which *Apc* mutations activate mTORC1 independently of and in parallel with β-catenin signaling to promote colorectal cancer development and progression. Combined inhibition of mTORC1 and Wnt signaling could prove to be an effective strategy for treating colorectal cancer. A role for mTORC1 in FAP also suggests parallels with other polyposis syndromes, including Peutz-Jeghers and Cowden’s syndrome, which are also associated with mTORC1 activation.

To investigate a role for mTORC1 downstream of APC, we have examined oncogenic *Apc* mutations in zebrafish and mice. Homozygous *Apc* mutant mice die before gastrulation (Fodde et al., 1994), whereas homozygous mutant zebrafish (*apc^mcr/mcr^* zebrafish) survive until early larval stages (Hurlstone et al., 2003). The *Apc* mutant zebrafish have multiple developmental defects including impaired circulation, hyperproliferation of the cardiac cushions, enlarged livers and defects in morphogenesis of the anterior-posterior axis, resulting in severe body curvature (Goessling et al., 2008; Hurlstone et al., 2003). These observations indicate a crucial role for APC in the development of many organs and tissues.

Here, we show that mTORC1 is aberrantly activated in *apc^mcr/mcr^* zebrafish and that mTORC1 inhibition attenuates multiple phenotypes in *Apc* mutants, indicating that mTORC1 activation is important for these phenotypes. We also find that mTORC1 is robustly activated in intestinal adenomas in *Apc^min^* mice. Taken together, these data suggest that mTORC1 is a crucial, β-catenin-independent effector that is activated by *Apc* mutation.

## RESULTS

### Oncogenic *Apc* mutation activates mTORC1 in zebrafish larvae

**Fig. 1.**
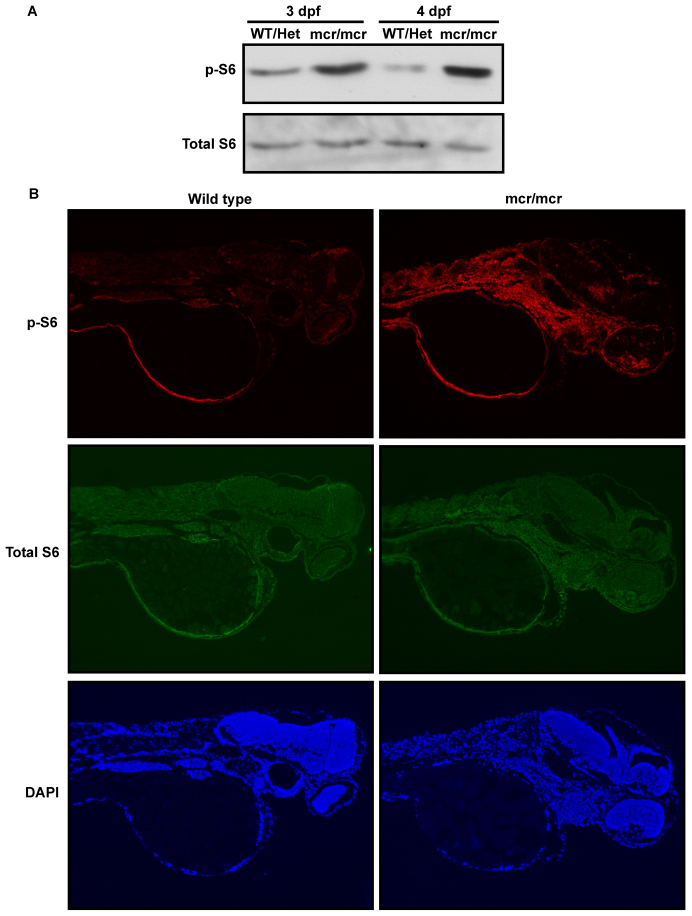
**Activation of mTORC1 in *apc^mcr/mcr^* zebrafish.** Western blot analysis using antibodies to phosphorylated S6 (p-S6), indicating mTORC1 activity, or total S6 on whole embryo lysates from pooled wild-type and heterozygous *apc^mcr/+^* zebrafish or homozygous *apc^mcr/mcr^* zebrafish at 3 or 4 dpf. mTORC1 activity was increased in the homozygous *apc^mcr/mcr^* zebrafish. (B) Immunohistochemical staining of sagittal sections from wild-type or homozygous *apc^mcr/mcr^* zebrafish at 3 dpf using antibodies to phosphorylated or total S6 and counterstaining with DAPI. mTORC1 was aberrantly active in multiple mesodermal and endodermal derivatives in the homozygous *apc^mcr/mcr^* zebrafish.

We previously found that APC directly enhances the kinase activity of GSK-3 (Valvezan et al., 2012). GSK-3 inhibits mTORC1 activity (Inoki et al., 2006) and thus knocking down APC reduces GSK-3 activity and activates mTORC1 in cultured cells (Valvezan et al., 2012). We therefore hypothesized that oncogenic APC mutations should activate mTORC1 *in vivo*. For these studies we used *apc^mcr^* zebrafish, which contain a previously characterized mutation that causes a premature stop codon within the mutation cluster region (mcr) of *apc*, analagous to truncating mutations typically found in human colorectal cancers (Haramis et al., 2006; Hurlstone et al., 2003). We compared mTORC1 activity in *apc^mcr/mcr^* zebrafish to that in wild-type and heterozygous zebrafish. Homozygous *Apc* mutation activates mTORC1, as assessed by western blotting of whole embryo lysates for phosphorylation of ribosomal protein S6, a well-established readout of mTORC1 activity (Laplante and Sabatini, 2012). Phosphorylated S6 is increased in homozygous mutants at 3 and 4 days post fertilization (dpf) compared with pooled wild-type and heterozygous fish, without a change in total S6 (Fig. 1A). To assess tissue-specific changes in mTORC1 activity, we also performed immunohistochemistry on larvae fixed at 3 dpf. Immunostaining for phosphorylated S6 reveals broadly increased mTORC1 activity in multiple mesodermal and endodermal derivatives (Fig. 1B). These data indicate that an oncogenic *Apc* mutation activates mTORC1.

### mTORC1 inhibition extends the survival of *apc^mcr/mcr^* zebrafish

**Fig. 2.**
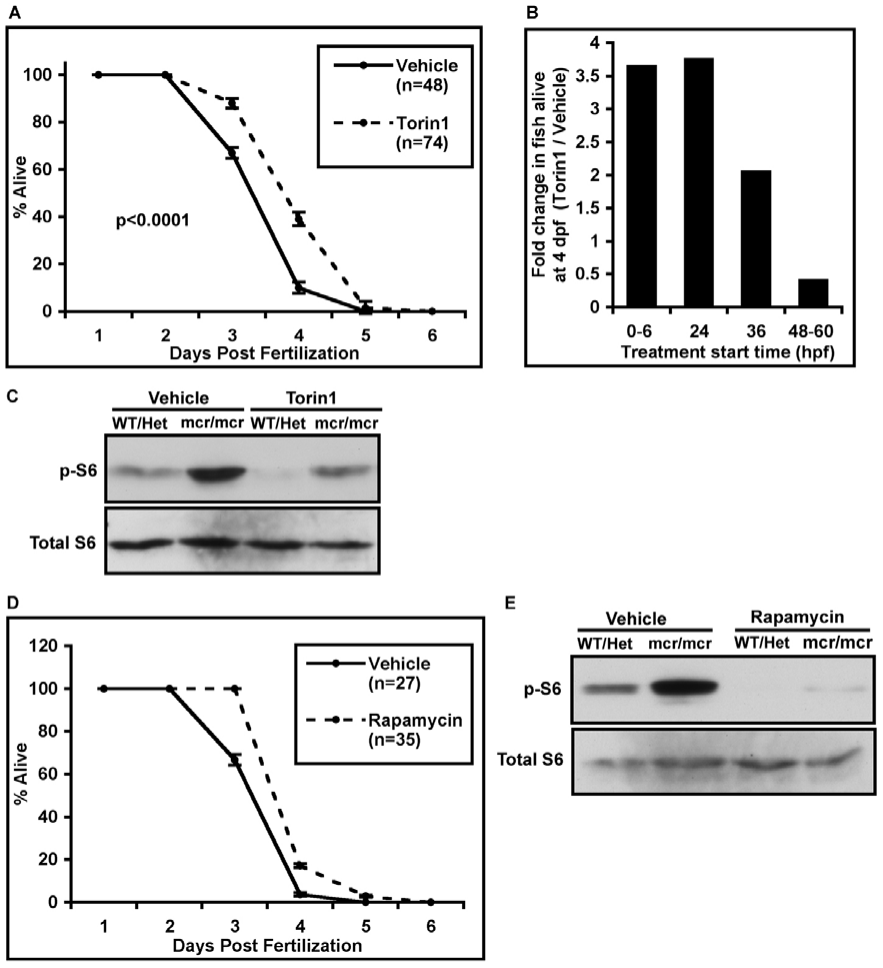
**mTORC1 inhibition extends survival in *apc^mcr/mcr^* zebrafish.** (A) *apc^mcr/mcr^* zebrafish were treated with vehicle or Torin1 (250 nM) starting at 24 hpf. The percentage of fish alive was recorded every 24 hours. *P-*value for comparison of Torin1 vs vehicle treatment was calculated using the log-rank (Mantel-Cox) test. (B) Torin1 (250 nM) was added between 0 and 6 hpf, at 24 hpf, at 36 hpf or between 48 and 60 hpf. The percentage of Torin1-treated fish alive at 4 dpf was normalized to the percentage of vehicle-treated fish alive at 4 dpf. (C) Western blot analysis using antibodies to phosphorylated S6 or total S6 on whole embryo lysates from pooled 3-day-old wild-type (WT) and heterozygous (Het) *apc^mcr/+^* zebrafish or homozygous *apc^mcr/mcr^* zebrafish treated with vehicle or Torin1. Torin1 treatment under these conditions (250 nM) reduced mTORC1 activity in homozygous *apc^mcr/mcr^* zebrafish to levels similar to those in vehicle-treated wild-type/heterozygous fish. (D) Survival curves of *apc^mcr/mcr^* zebrafish treated with vehicle or rapamycin (200 nM) starting at 24 hpf. (E) Western blot analysis, as in C, on fish treated with vehicle or rapamycin. Error bars represent s.e.m.

To test whether aberrantly active mTORC1 contributes to *apc^mcr/mcr^* phenotypes, we asked whether mTORC1 inhibition rescues phenotypes in these fish. Torin1, a direct inhibitor of mTOR kinase activity (Thoreen et al., 2009), was added to developing zebrafish embryos at 24 hours post fertilization (hpf) and survival was assessed daily. Vehicle-treated *apc^mcr/mcr^* zebrafish larvae died 3-4 days post fertilization (dpf) whereas treatment with Torin1 extends their survival by up to 24 hours, with ~40% of Torin1-treated larvae surviving at 4 dpf compared with ~10% of vehicle-treated controls (*P*<0.0001; Fig. 2A). We also assessed the effect of adding Torin1 at different developmental stages. Adding Torin1 at 0–6 hpf or 24 hpf had similar effects on survival: Approximately 3.5 times as many Torin1-treated *apc^mcr/mcr^* zebrafish were alive at 4 dpf compared with vehicle-treated *apc^mcr/mcr^* zebrafish when treatment was started at either 0–6 hpf or 24 hpf (Fig. 2B). Treatment at 36 hpf or later was less effective (Fig. 2B). Importantly, Torin1 treatment reduced mTORC1 activity in homozygous *apc^mcr/mcr^* zebrafish to levels similar to those in vehicle-treated wild-type/heterozygous fish (Fig. 2C), highlighting the role for aberrant mTORC1 activation resulting from oncogenic *Apc* mutation. To address whether the effects of Torin1 are specifically mediated by mTORC1 inhibition, we also treated *apc^mcr/mcr^* zebrafish with rapamycin, an allosteric inhibitor of mTORC1 that acts through a distinct mechanism and through a separate binding site in the mTORC1 complex (Sarbassov et al., 2006). Rapamycin similarly extends survival of *apc^mcr/mcr^* zebrafish (Fig. 2D). These data suggest that aberrant mTORC1 activation resulting from oncogenic *Apc* mutation contributes to early lethality in *apc^mcr/mcr^* zebrafish.

### mTORC1 inhibition improves circulation in *apc^mcr/mcr^* zebrafish

**Fig. 3.**
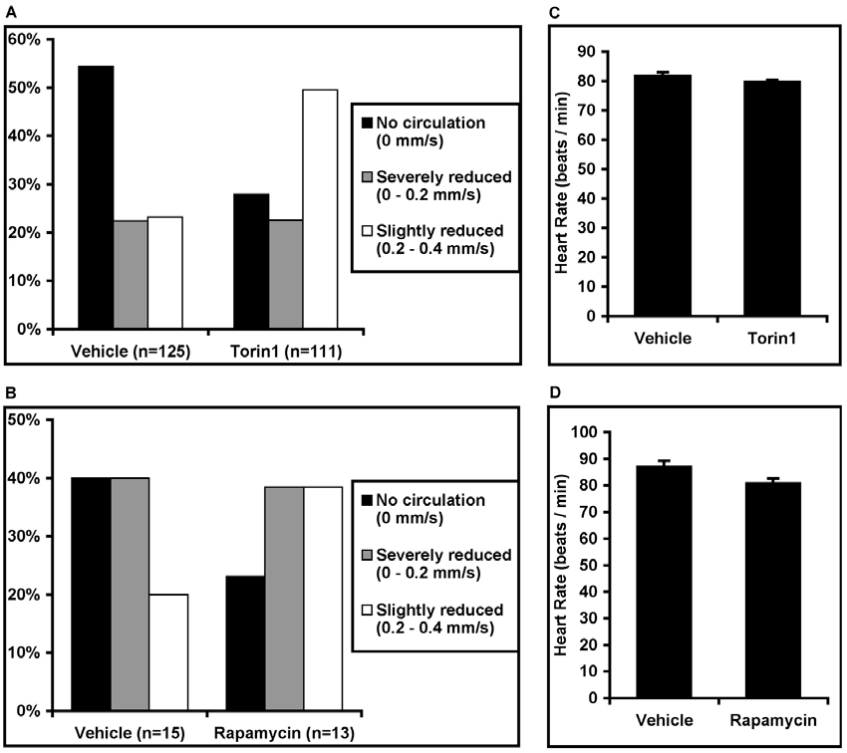
**mTORC1 inhibition improves circulation in *apc^mcr/mcr^* zebrafish.** (A) *apc^mcr/mcr^* zebrafish were treated with vehicle or Torin1 (250 nM) and the rate of blood flow through the dorsal aorta was measured using high speed videomicroscopy in unanesthetized larvae at 2–3 dpf. Blood flow phenotypes were categorized according to flow rate as indicated. Representative movies for each scoring category can be found in online supplementary material (supplementary material Movies 1–4). (B) Rapamycin also improved circulation in *apc^mcr/mcr^* zebrafish. (C,D) Torin1 and rapamycin treatment did not increase the heart rate of *apc^mcr/mcr^* zebrafish.

The *apc^mcr/mcr^* zebrafish have dramatically reduced circulation compared with wild-type fish at 2–3 dpf (supplementary material Movies 1–4) (Hurlstone et al., 2003). We used high speed videomicroscopy to measure blood flow through the dorsal aorta; approximately half of vehicle-treated fish had no circulation through the dorsal aorta and ~75% had a flow rate of less than 0.2 mm/second (Fig. 3A). Torin1 treatment improves circulation, resulting in a flow rate greater than 0.2 mm/second in ~50% of mutants, and reducing the percentage of mutants with no circulation to ~25% (Fig. 3A). Rapamycin similarly doubled the percentage of mutants with flow greater than 0.2 mm/second and halved the percentage of mutants with no circulation (Fig. 3B). Representative movies showing blood flow through the dorsal aorta in wild-type fish and each of the three categories used for scoring *apc^mcr/mcr^* zebrafish can be found in supplementary material Movies 1–4. Torin1 and rapamycin did not increase the heart rate of the *apc^mcr/mcr^* zebrafish (Fig. 3C,D). These data indicate that mTORC1 activation in *apc^mcr/mcr^* zebrafish contributes to the reduced circulation phenotype.

### mTORC1 inhibition rescues liver hyperplasia in heterozygous *apc^mcr/+^* zebrafish

**Fig. 4.**
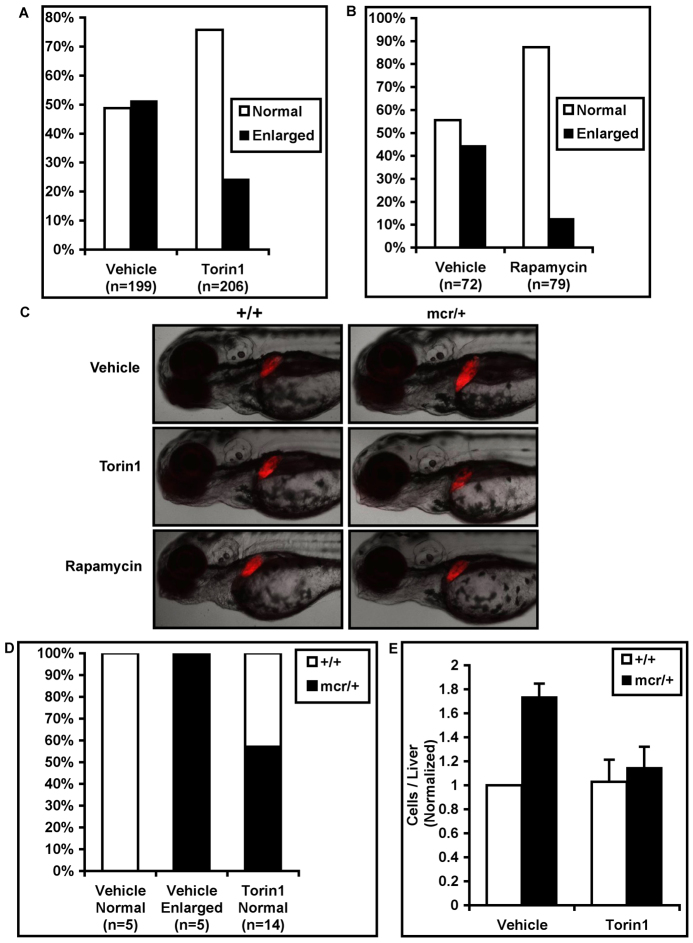
**mTORC1 inhibition rescues liver hyperplasia in heterozygous *apc^mcr/+^* zebrafish.** *Lfabp:rfp* zebrafish, which express liver-specific RFP, were mated with *apc^mcr/+^* zebrafish. (A) Resulting progeny (expected 50% *apc^+/+^*, 50% *apc^mcr/+^*) were treated with vehicle or Torin1 and the percentage of fish with normal or enlarged livers at 3–4 dpf was recorded. Approximately 50% of vehicle-treated fish had enlarged livers, as described previously (Goessling et al., 2008). Torin1 reduced the percentage of fish with enlarged livers. (B) Rapamycin similarly rescued liver enlargement. (C) Representative pictures showing liver enlargement in vehicle-treated heterozygous *apc^mcr/+^* zebrafish but not in Torin1- or rapamycin-treated heterozygotes. (D) Liver size correlated closely with genotype in vehicle-treated embryos, as described previously (Goessling et al., 2008), but heterozygous *apc^mcr/+^* embryos accounted for approximately half of Torin1-treated embryos with normal-size livers, confirming that Torin1 reduces liver size in heterozygous *apc^mcr/+^* embryos. (E) The number of RFP-positive cells per embryo was measured by flow cytometry. Vehicle-treated *apc^mcr/+^* zebrafish had ~75% more hepatocytes than vehicle-treated wild-type fish, and this was rescued by Torin1 treatment.

Heterozygous *apc^mcr/+^* zebrafish had enlarged livers by 3 dpf due to an increase in the number of hepatocytes (Fig. 4) (Goessling et al., 2008). To investigate whether this phenotype can be rescued by mTORC1 inhibition, we crossed heterozygous *apc^mcr/+^* zebrafish to *lfabp:RFP* fish, which express red fluorescent protein under control of the liver-specific liver fatty acid binding protein (lfabp) promoter (Her et al., 2003). About 50% of resulting progeny are expected to be heterozygous *apc^mcr/+^* and 50% are expected to be wild type at the *Apc* locus (*apc^+/+^*). We scored liver size in the resulting RFP+ progeny and found that ~50% had normal-size livers and 50% had enlarged livers, as previously reported (Goessling et al., 2008). Treatment with Torin1 or rapamycin greatly reduced the percentage of fish with enlarged livers and increased the percentage with normal livers (Fig. 4A,B). Genotyping confirmed a strong correlation between heterozygous *Apc* mutation and liver enlargement in vehicle-treated fish (Fig. 4C,D). However, when Torin1-treated fish with normal-size livers were genotyped, approximately half were found to be *apc^mcr/+^*, confirming that Torin1 restores normal liver size in heterozygous *Apc* mutant zebrafish (Fig. 4C,D). To quantify the effect of Torin1 treatment on liver hyperplasia, the number of RFP-positive cells per liver was measured by flow cytometry. The *apc^mcr/+^* fish had ~75% more cells per liver and this was restored to wild-type levels by Torin1 (Fig. 4E). Torin1 had no effect on the number of hepatocytes in wild-type zebrafish. These data demonstrate a crucial role for mTORC1 in liver hyperplasia resulting from oncogenic *Apc* mutation.

### Combined inhibition of mTORC1 and Wnt signaling reduces body curvature in *apc^mcr/mcr^* zebrafish

**Fig. 5.**
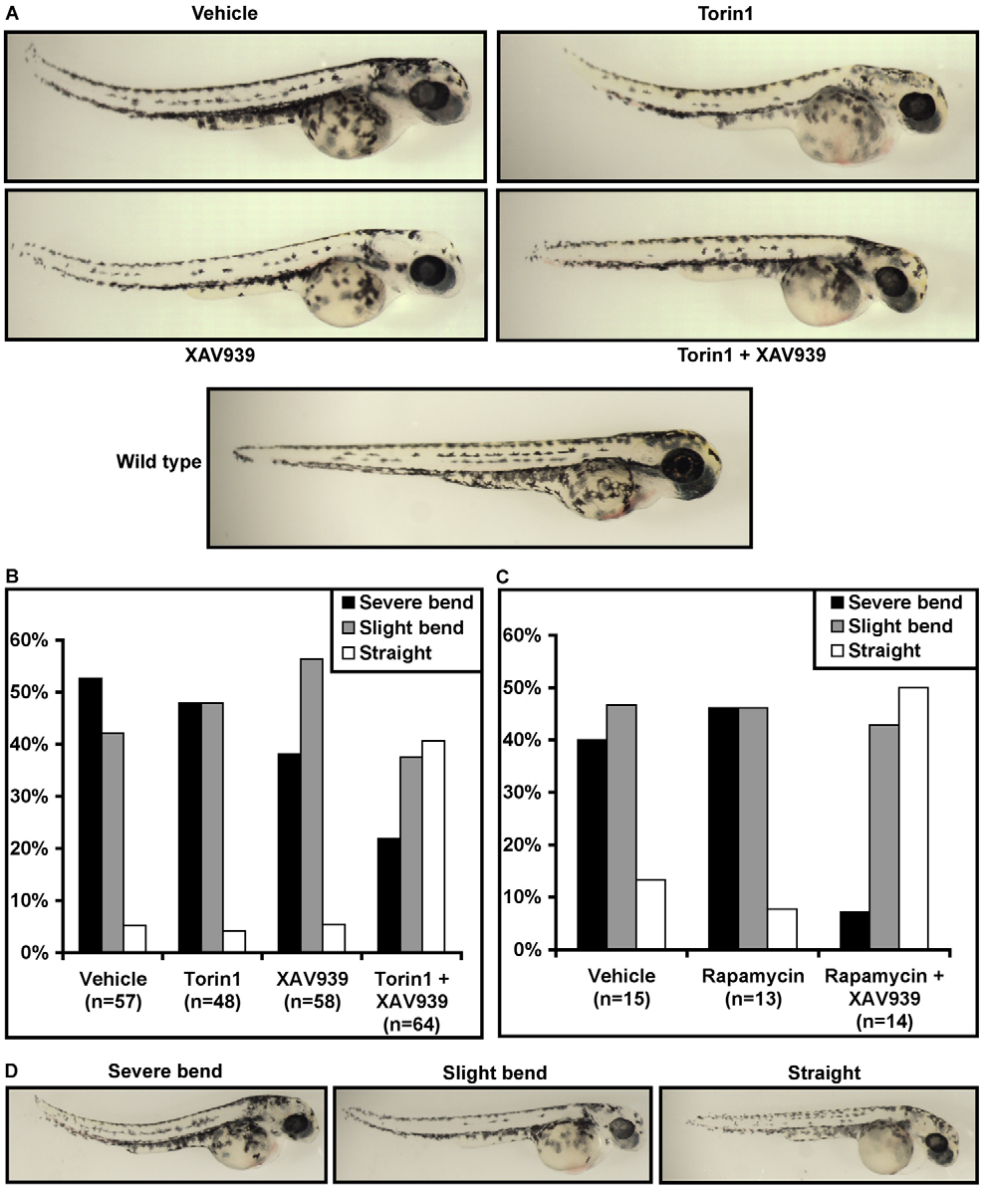
**Combined inhibition of mTORC1 and Wnt signaling reduces body curvature in *apc^mcr/mcr^* zebrafish.** (A) Representative images of *apc^mcr/mcr^* zebrafish at 2 dpf treated with vehicle, Torin1 (250 nM), XAV939 (500 nM) or Torin1 plus XAV939. Combined Torin1 and XAV939 treatment rescued body curvature whereas either treatment alone did not. (B) Zebrafish were scored at 2-3 dpf on the basis of severity of body curvature; the percentage of fish in each category is shown. Only combined Torin1 plus XAV939 treatment reduced body curvature. (C) Rapamycin also reduced body curvature in combination with XAV939, but not by itself. (D) Representative pictures of each of the three categories used to score body curvature in B and C.

We have found that aberrant mTORC1 activation contributes to early lethality, reduced circulation and liver hyperplasia resulting from oncogenic *Apc* mutation. *Apc* loss of function also activates Wnt/β-catenin signaling, which plays a major role in *Apc* mutant phenotypes. Thus we asked whether combined inhibition of mTORC1 and Wnt/β-catenin signaling could rescue additional phenotypes. Defective morphogenesis of the anterior-posterior axis resulting in aberrant body curvature is one of the most prominent phenotypes in *apc^mcr/mcr^* zebrafish (Fig. 5) (Hurlstone et al., 2003). Treatment with either Torin1 or the Wnt inhibitor XAV939 (Huang et al., 2009b) alone did not affect body curvature, but combining Torin1 and XAV939 resulted in *apc^mcr/mcr^* zebrafish with straight bodies similar to wild type (Fig. 5A). Approximately 95% of vehicle-, Torin1- or XAV939-treated *apc^mcr/mcr^* zebrafish had curved body axes, whereas ~40% of *apc^mcr/mcr^* zebrafish treated with both Torin1 and XAV939 were straight (Fig. 5B). Similarly, rapamycin improved body curvature in combination with XAV939 but had no effect on its own (Fig. 5C). Representative images of scoring categories used in Fig. 5B and 5C are shown in 5D. These data suggest that *Apc* mutation activates mTORC1 and Wnt/β-catenin signaling independently and highlight the importance of their combined activation. Furthermore, these findings demonstrate that simultaneous inhibition of both pathways can rescue *Apc* mutant phenotypes that are not rescued by targeting either pathway alone.

### mTORC1 is strongly activated in *Apc^min^* mouse intestinal polyps

**Fig. 6.**
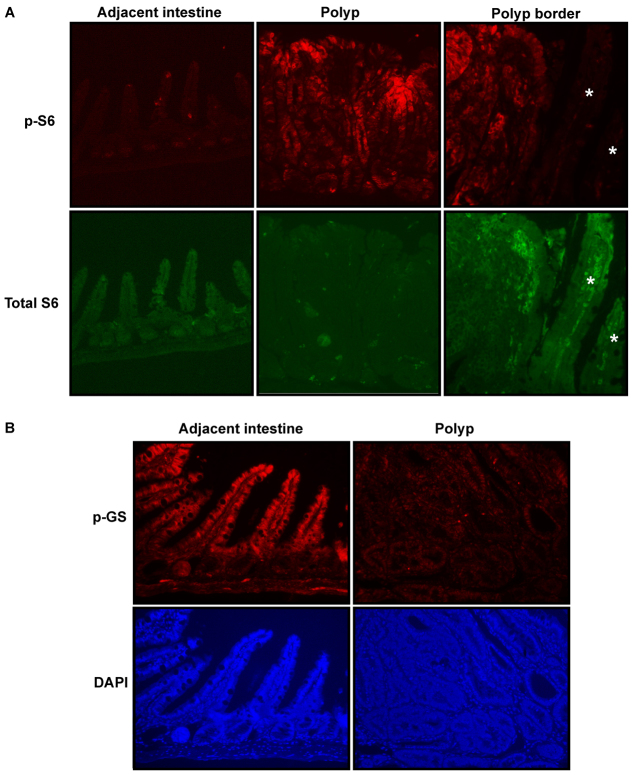
**mTORC1 is strongly activated and glycogen synthase phosphorylation is reduced in *Apc^min^* mouse intestinal polyps.** Immunostaining of intestinal sections from *Apc^min^* mice. (A) Staining for phosphorylated S6, indicating mTORC1 activity, or total S6 revealed that mTORC1 activity was dramatically increased in polyps compared with immediately adjacent normal tissue. Asterisks indicate normal villi immediately adjacent to a polyp. (B) Immunostaining using an antibody to glycogen synthase phosphorylated at the GSK-3 phosphorylation site and counterstained with DAPI. Glycogen synthase phosphorylation was strongly reduced in polyps compared with immediately adjacent normal intestine.

To confirm our findings in zebrafish using an established mammalian model of polyposis, we examined GSK-3 and mTORC1 activity in *Apc^min^* mice, which develop intestinal adenomas through loss of heterozygosity (LOH) for *Apc*. Phospho-S6 staining was strongly induced in polyps compared with adjacent normal tissue (Fig. 6A), similar to previous reports (Fujishita et al., 2008; Metcalfe et al., 2010). Furthermore, phosphorylation of glycogen synthase, an endogenous GSK-3 substrate, was markedly reduced in polyps, but not normal epithelium, from *Apc^min^* mice (Fig. 6B). These data are consistent with a model in which loss of APC reduces GSK-3 activity, which in turn activates both mTORC1 and Wnt/β-catenin signaling.

## DISCUSSION

The importance of enhanced Wnt/β-catenin activity as a mediator of *Apc* loss-of-function phenotypes is firmly established from studies in model organisms and in colorectal carcinomas in humans. However, reports that early neoplastic lesions can appear before nuclear localization of β-catenin is detectable have raised the possibility that APC regulates additional effectors that contribute to *Apc* mutant phenotypes in parallel with, but independent of, nuclear β-catenin. We show that truncating mutations in *Apc*, which are homologous to those found in human colorectal cancers and FAP, cause marked activation of mTORC1 and that mTORC1 activation is important for the resulting phenotypes, including lethality in the larva stage, impaired circulation, defects in morphogenesis and liver hyperplasia. These findings support a crucial role for mTORC1 downstream of oncogenic *Apc* mutations.

These findings are consistent with a signaling pathway in which APC directly enhances GSK-3 activity (Valvezan et al., 2012). We previously showed that APC directly facilitates GSK-3 activity *in vitro* and that knockdown of APC reduces GSK-3 activity toward endogenous substrates, including glycogen synthase, in cultured cells. Multiple functional parallels between APC and GSK-3 support this model. Thus, APC and GSK-3 play similar roles in bone morphogenic protein (BMP) signaling (Fuentealba et al., 2007; Fukuda et al., 2010; Miclea et al., 2011), ERK signaling (Park et al., 2006; Wang et al., 2006; Zhai et al., 2007), mitosis (Dikovskaya et al., 2012; Hanson and Miller, 2005; Happel et al., 2009; Wakefield et al., 2003), stem cell homeostasis (Huang et al., 2009a; Qian et al., 2008) and embryonic development in *C. elegans* (Rocheleau et al., 1997; Schlesinger et al., 1999), *Drosophila* (McCartney et al., 2001; Yu et al., 1999) and zebrafish (Hurlstone et al., 2003; Lee et al., 2007). Inoki et al. showed that GSK-3 suppresses mTORC1 activity (Inoki et al., 2006), and this was confirmed *in vivo* in mouse bone marrow (Huang et al., 2012; Huang et al., 2009a); thus our model predicts that inhibition of GSK-3 caused by *Apc* loss of function should activate mTORC1 *in vivo*. We observed that glycogen synthase phosphorylation is substantially reduced in intestinal polyps from *Apc^min^* mice (Fig. 6B), which supports our model that *Apc* mutation reduces GSK-3 activity. We also confirm that mTORC1 is markedly activated in *Apc* mutant polyps (Fig. 6A), consistent with previous reports (Fujishita et al., 2008; Metcalfe et al., 2010). Furthermore, mTORC1 inhibitors reduce adenoma size and number, as well as mortality, in *Apc* mutant mice, demonstrating the importance of mTORC1 activation for tumorigenesis resulting from *Apc* mutation (Fujishita et al., 2008; Koehl et al., 2010; Metcalfe et al., 2010).

In contrast to adult *Apc^min/+^* mice [as well as adult *apc^mcr/+^* zebrafish (Haramis et al., 2006; Hurlstone et al., 2003)], the intestines in *apc^mcr/mcr^* homozygous mutant larvae develop with reduced cell number, disorganization of the epithelium and narrowing of the intestinal lumen (Faro et al., 2009; Phelps et al., 2009b). Neither Torin1 nor rapamycin treatment rescued this phenotype (data not shown), perhaps because the defect is not associated with increased proliferation and/or because other factors independent of mTORC1 activation play a key role in this mutant phenotype.

Previous studies have suggested that mTOR expression is increased by Wnt/β-catenin signaling (Fujishita et al., 2008; Metcalfe et al., 2010). However, our data suggest that APC also regulates mTORC1 by enhancing GSK-3 activity independently of β-catenin (Figs 5, 6) (Valvezan et al., 2012); although these two mechanisms are distinct, they are not exclusive of each other. Nevertheless, our observations further suggest that experimental manipulations of upstream Wnt pathway components do not necessarily give information on β-catenin function because Wnts, APC, Axin and GSK-3 also regulate mTORC1 (Fig. 1) (Inoki et al., 2006; Valvezan et al., 2012). Thus, although results from previous studies examining the effects of Dkk-1 on zebrafish cardiac development (Hurlstone et al., 2003) and Dvl2 in mouse intestine (Metcalfe et al., 2010) support a role for canonical Wnt signaling, they do not distinguish between effects mediated by β-catenin and mTORC1.

The importance of combined Wnt/β-catenin and mTORC1 signaling is also supported by our finding that aberrant body curvature in *apc^mcr/mcr^* zebrafish is rescued by combining mTORC1 inhibition with a Wnt pathway inhibitor, as neither inhibitor alone achieves rescue. We find that hyperplasia of the liver in heterozygous *apc^mcr^* zebrafish is partially rescued by mTORC1 inhibition, and previous work has demonstrated that this phenotype is also partially rescued by β-catenin knockdown (Goessling et al., 2008), again indicating a role for both β-catenin and mTORC1 in this hyperplastic phenotype. Taken together, these data suggest that activation of both mTORC1 and Wnt/β-catenin signaling contributes to multiple phenotypes resulting from oncogenic *Apc* mutation.

In further support of a role for mTORC1 downstream of APC, *apc^mcr^* zebrafish are partially phenocopied by loss of function in TSC components: thus knockdown of *Tsc1* activates mTORC1 and causes similar defects in body curvature and genetic inactivation of *Tsc2* similarly activates mTORC1, results in early lethality and causes liver hyperplasia similar to *apc^mcr^* heterozygotes (DiBella et al., 2009; Goessling et al., 2008; Hurlstone et al., 2003; Kim et al., 2011). Importantly, liver hyperplasia in *Tsc2* mutants is rescued by rapamycin, suggesting that the hyperplasia is caused by mTORC1 activation. The *Tsc2* phenotype is not identical to *apc^mcr^*, however. This could be due to persistence of maternal *Tsc2* gene expression relative to *Apc* as well as the important role of β-catenin and possibly other effectors downstream of APC.

Extracolonic manifestations in FAP include polyps of the gastric fundus and small bowel, osteomas, lipomas, desmoid tumors, adrenal cortical adenomas, congenital hypertrophy of the retinal pigment epithelium (CHRPE) and hepatoblastomas (Rustgi, 2007), suggesting parallels with other hereditary disorders that cause neoplasias in multiple organs, including tuberous sclerosis, Peutz-Jeghers syndrome and Cowden’s syndrome (Crino et al., 2006; Inoki et al., 2005; Rustgi, 2007). Peutz-Jeghers and Cowden’s syndromes are hamartomatous polyposis syndromes that involve multiple organ systems and are mediated in part through activation of mTORC1. Peutz-Jeghers syndrome is caused by mutations in LKB, an upstream regulator of AMPK and TSC2, and Cowden’s syndrome is caused by mutations in PTEN that also activate mTORC1 (Inoki et al., 2005). Although each of these syndromes is clinically and pathologically distinct, it is interesting to note that they all share mTORC1 activation, which might be an early molecular step in tumor formation in each case. This parallel also suggests that mTORC1 inhibitors might be effective in controlling some of the extracolonic manifestations of FAP. In contrast, the differences in these syndromes could be due to the fact that APC, LKB and PTEN each regulate multiple other effectors.

In conclusion, APC negatively regulates mTORC1 and oncogenic *Apc* mutations disrupt this function, resulting in constitutive mTORC1 activation in both zebrafish and mammalian models. Several resulting phenotypes are partially rescued by mTORC1 inhibition, demonstrating a widespread role for mTORC1 activation downstream of APC mutation. Combined inhibition of mTORC1 and Wnt signaling rescues additional phenotypes, highlighting the importance of the unique combination of active mTORC1 and Wnt that results from oncogenic APC mutation. Combined inhibition of Wnt and mTORC1 signaling could be an effective strategy for treatment of colorectal cancers resulting from *Apc* mutation.

## MATERIALS AND METHODS

### Zebrafish

Embryos were raised at 28.5°C in standard E3 medium (Westerfield, 1993). Pronase (30 μg/ml; Roche #10165921001) was added at 24 hpf to digest the chorion and washed out at 48 hpf. The *apc^mcr^* zebrafish and primers used for genotyping were described previously (Hurlstone et al., 2003). *Lfabp:rfp* zebrafish were described previously (Her et al., 2003). Rapamycin (LC Laboratories #R-5000), Torin1 (Tocris Bioscience #4247) and/or XAV939 (Sigma #X3004) were added directly into the fish medium starting at 24 hpf unless otherwise indicated. Medium was changed and fresh treatments were added every 24 hours. Videomicroscopy and quantification of blood flow rate was performed in unanesthetized zebrafish larvae at 60 hpf as previously described (Hoage et al., 2012). Flow cytometry was performed as previously described (Goessling et al., 2008) except that embryos were dissociated in 0.25% trypsin (Westerfield, 1993). For immunofluorescence, 3-dpf zebrafish embryos were fixed in 4% paraformaldehyde for 2 hours at room temperature, washed in PBST, dehydrated in an ascending ethanol series and submitted to the Perelman School of Medicine Cancer Histology Core at the University of Pennsylvania for paraffin embedding and sectioning. Immunofluorescence staining for S6 and phospho-S6 was performed as in *Apc^min^* mouse intestinal sections (see below). Zebrafish husbandry and egg procurement were carried out in accordance with the guidelines of the University of Pennsylvania Institutional Animal Care and Use Committee.

### Lysis and western blots

Zebrafish embryos were lysed on ice in 5 μl/embryo of buffer containing 1% NP-40, 20 mM Tris pH 8.0, 50 mM NaCl, 2.5 mM EDTA, 1 mM DTT, protease inhibitor cocktail (Sigma #P8340) diluted 1:100, phosphatase inhibitor cocktails #2 (Sigma #P5726) and #3 (Sigma #P0044) also diluted 1:100 each. An equal volume of standard 2× Laemmli Sample Buffer was added and samples were heated at 95°C for 5 minutes and then centrifuged at 14,000 rpm for 5 minutes at 4°C. Supernatants were collected for SDS-PAGE and western blot analysis using the following antibodies purchased from Cell Signaling Technology: S6 ribosomal protein (#2317) and phospho-S6 ribosomal protein Ser235/236 (#4858).

### Apc^min^ mice

*Apc^min^* mice were purchased from The Jackson Laboratory (stock #002020) and have been described previously (Moser et al., 1993). For immunofluorescence, intestines were fixed in 10% phosphate-buffered formalin overnight, washed in PBST, dehydrated in an ascending ethanol series and submitted to the Perelman School of Medicine Molecular Pathology and Imaging Core at the University of Pennsylvania for paraffin embedding and sectioning. Paraffin-embedded sections were washed in xylene and rehydrated in a descending ethanol series followed by boiling in 10 mM sodium citrate pH 6.0 and blocking in Starting Block buffer (Thermo Scientific #37539). Antibodies to S6, phospho-S6 Ser235/236 or phospho-glycogen synthase Ser641 (Cell Signaling Technology #3891) were added overnight at 4°C followed by incubation with Cy2- or Cy3-conjugated secondary antibodies.

## Supplementary Material

Supplementary Material
