# Supplementary material for: Oncogenic mutations in adenomatous polyposis coli (Apc) activate mechanistic target of rapamycin complex 1 (mTORC1) in mice and zebrafish
Source: Dis Model Mech. 2013 Oct 2;7(1):63–71. doi: 10.1242/dmm.012625 (PMC3882049; doi:10.1242/dmm.012625)
Supplement: Supplementary Material [file supp_7_1_63__index.html]

Oncogenic mutations in adenomatous polyposis coli (Apc) activate mechanistic target of rapamycin complex 1 (mTORC1)in mice and zebrafish — Oncogenic mutations in adenomatous polyposis coli (Apc) activate mechanistic target of rapamycin complex 1 (mTORC1) in mice and zebrafish — Supplementary Material 

# Oncogenic mutations in adenomatous polyposis coli (*Apc*) activate mechanistic target of rapamycin complex 1 (mTORC1) in mice and zebrafish

## DMM012625 Supplementary Material

**Files in this Data Supplement:**

- **Supplementary Material PDF**
