# Supplementary material for: Oncogenic mutations in adenomatous polyposis coli (Apc) activate mechanistic target of rapamycin complex 1 (mTORC1) in mice and zebrafish
Source: Dis Model Mech. 2013 Oct 2;7(1):63–71. doi: 10.1242/dmm.012625 (PMC3882049; doi:10.1242/dmm.012625)
Supplement: Supplementary Material [file supp_012625_DMM012625.pdf]

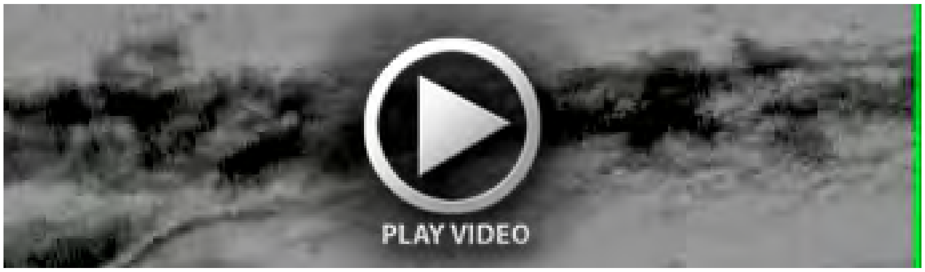

Movie 1. Wild type circulation. Representative video showing circulation through the dorsal aorta of a wild type zebrafish at ~60 hpf. Circulation is rapid (mean flow rate >0.4 mm/sec) and pulsatile.

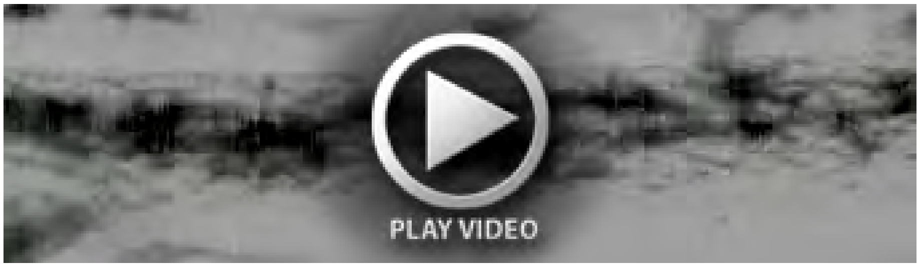

Movie 2. Slightly reduced circulation. Representative video showing circulation through the dorsal aorta of an $apc^{mcr/mcr}$ zebrafish scored as "Slightly reduced" in Fig. 3 at ~60 hpf. Mean flow for this group was 0.2-0.4 mm/sec and was still pulsatile.

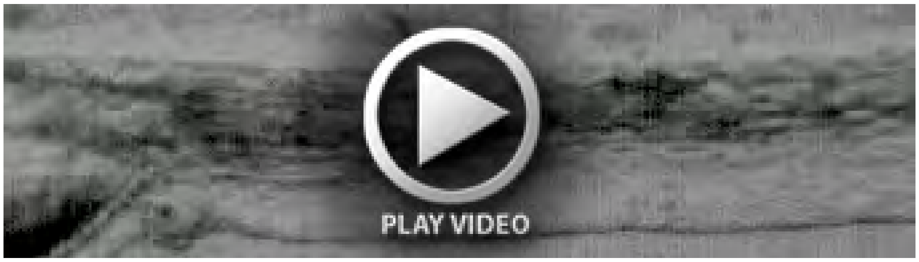

Movie 3. Severely reduced circulation. Representative video showing circulation through the dorsal aorta of an $apc^{mcr/mcr}$ zebrafish scored as "Severely reduced" in Fig. 3 at ~60 hpf. This category was defined as mean flow rate of 0-0.2 mm/sec.

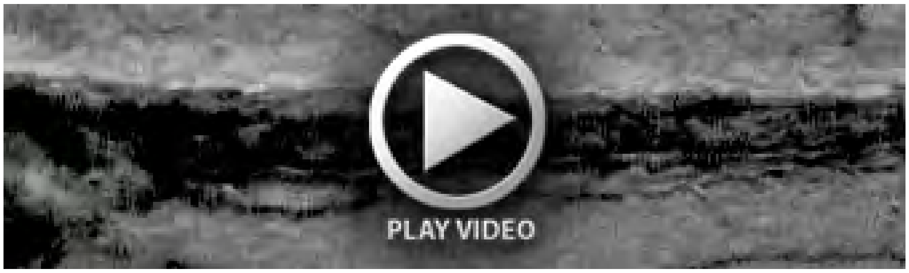

Movie 4. No circulation. Representative video showing lack of circulation through the dorsal aorta of an $apc^{mcr/mcr}$ zebrafish scored as having "No circulation" in Fig. 3 at ~60 hpf. No circulating cells are visible.
